# Synthesis in Silica Nanoreactor: Copper Pyrophosphate Quantum Dots and Silver Oxide Nanocrystallites Inside Silica Mezochannels

**DOI:** 10.3390/ma13092009

**Published:** 2020-04-25

**Authors:** Łukasz Laskowski, Anna Majtyka-Piłat, Krzysztof Cpałka, Maciej Zubko, Magdalena Laskowska

**Affiliations:** 1 Institute of Nuclear Physics Polish Academy of Sciences, PL-31342 Krakow, Poland; lukasz.laskowski@ifj.edu.pl; 2 Silesian Center for Education and Interdisciplinary Research, Institute of Materials Science, Faculty of Computer Science and Materials Science, University of Silesia, ul. 75 Pułku Piechoty 1A, 41-500 Chorzów, Poland; anna.majtyka@us.edu.pl (A.M.-P.); maciej.zubko@us.edu.pl (M.Z.); 3 Institute of Computational Intelligence, Czestochowa University of Technology, 42-200 Czestochowa, Poland; krzysztof.cpalka@iisi.pcz.pl; 4 Department of Physics, Faculty of Science, University of Hradec Králové, Rokitanského 62, 500 03 Hradec Králové, Czech Republic

**Keywords:** functional materials, mesoporous silica, nanocrystals, confinements, nanoreactors

## Abstract

The synthesis routes are presented for the preparation of nanocomposites composed of nanocrystals placed inside SBA-15 silica pores. The procedures assume treating the silica channels as nanoreactors, where nanocrystals are created as a result of thermal decomposition of internal functional units. Its sizes and chemical composition can be modified by the change of functional group types and density inside silica channels. The procedure is demonstrated by the example of copper pyrophosphate quantum dots and silver oxide nanoparticles inside silica mezochannels. The method can be easily adopted to other types of nanocrystals that can be synthesized inside silica nanoreactors.

Nanocrystals can find numerous applications in modern technology. Catalysis, nanophotonics and medicine can be mentioned as the most important.

For the catalysis, one of the most important factors increasing the catalytic activity of the specimen is the active surface area of material [1]. For this reason, there are many efforts for obtaining relatively small catalytic moieties [2,3,4].

Similarly, in nanophotonics, the crystal size if a critical parameter which affects the optical response of material [5]. Nanocrystals can be an alternative for rare-earth elements is the color-convertor phosphors used in white LEDs [6]. Scientists propose treating the semiconducting nanocrystals as fluorescent colloidal nanocrystal quantum dots (NQDs) that can be used as wavelength-upconverting coatings onto blue LED chips being able to generate visible light via photoluminescence [7]. Nanocrystals can also be used as frequency transformers in lasers since nanocrystals are extremely effective as far as a third- and second-order harmonic generation are concerned [8,9].

In medicine, nanoparticles (NPs) and nanocrystals are used as biocidal agents. Especially gold, silver, and copper NPs are in everyday use [10,11], but also silver oxide can be used as a disinfectant agent [12,13]. Similarly, in this case, size is a key factor [14].

As far as catalysis and medicine are concerned, the most important factor is the active surface area of specimen. Preparing small and relatively uniform nanocrystals, nevertheless, is still a challenging task. To this aim, many ways can be developed. But how to keep the nanocrystals separated in such a way to harness its active surface area? How to separate active molecules or crystals from each other? There were some attempts to using solvents [15,16] or covering by the passive shells [17]. However, the most reasonable way seems to be using some ambient matrix for the active moieties [18,19]. Here, nevertheless, we deal with a few problems connected with assuring the homogenous distribution of the nanoobjects inside the matrix and maintaining the nanocomposite functions. Using the mesoporous silica matrix can help in overcoming these problems. This material is harmless for mammal tissues, including laser and it is non-soluble in the most of solvents, excepting HF. Its porosity gives a huge specific surface area for placing functional groups and assures access to them. In this case, the crucial is a proper synthesis procedure allowing for homogenous placing the nanocrystals inside silica pores. Considering the pore size in the most common porous silicas being 2 nm for MCM-41 [20] and 5 nm for SBA-15 [21], the homogenous distribution of the nanocrystals inside pores is not an easy task.

Here, a bottom-up approach is proposed to this task: the synthesis of some specific nanocrystals directly inside silica pores that are treated here as nanoreactors. A somewhat similar solution was applied for the fabrication of the metal nanowires [22,23]. In the mentioned cases, however, there was no possibility for the control of sizes of the internal nanostructures, nor its distribution. The method proposed by our team allows for obtaining small nanocrystals distributed roughly homogeneously inside pores with the possibility of the control of their size and chemical composition.

The idea of nanocrystal fabrication in silica nanoreactors is based on the thermal decomposition of the initial compound—precisely functionalized mesoporous silica (SBA-15 in this case). Such an initial material has some selected functional units homogeneously distributed inside silica pores. Therefore, the silica channels play the role of reactors, where the atomic composition of the “reagents” can be modified by the proper functional unit selection. Moreover, adjusting the doping rate enables the number of reagents to be set. This modification, in turn, influences the nanocrystal size inside pores. It is worth noting that the functional units in the initial specimen should be designed in such a way that they undergo thermal decomposition at selected temperature. In the next step, the sample is calcined, and the desired nanocrystals are obtained inside silica pores. The silica mezochannels create spatial confinement preventing from creation nanocrystals with a diameter larger than 5 nm (of course, one dimension can be greater than 5 nm, in this case, crystals can have a shape of a rod). The method is schematically depicted in Figure 1. To the best of our knowledge, this is the first time this solution is proposed and demonstrated.

To test the procedure, we fabricate two types of nanocomposites:

- copper pyrophosphate nanocrystals inside the SBA-15 silica pores and
- silver oxide nanocrystals also inside the SBA-15 channels.

The former specimen is essential for nanophotonics (can be used as a frequency converter in lasers). At the same time, the later material possesses strongly biocidal properties and can be applied for disinfection (the properties of the mentioned nanocomposites will be described in separate papers).

The materials were prepared in a powder-like form to demonstrate the technology and to prove the correctness of our assumption. However, the procedure can also be applied for a thin-film form of the silica.

The synthesis of materials can be divided into two stages:

- **stage 1**: the synthesis of the initial material—SBA-15 mesoporous silica possessing homogenously distributed functional units inside the pores;
- **stage 2**: thermal decomposition of the initial material, leading to the formation of nanocrystals inside the pores.

Both demonstration materials were prepared in a similar way. In the first stage, we applied the direct-synthesis method—co-condensation of the silica precursors for obtaining SBA-15 silica containing proper functional units. Unlike grafting or impregnation, in the direct-synthesis procedure, it is possible to control the distribution of the functional units inside silica channels. Moreover, this method allows for obtaining the homogenous distribution of functionalities. By selecting the appropriate functional groups, we can play with the atomic composition of the “reagents” inside the nanoreactor and obtain desired nanocrystals. At the same time, modifying the doping rate enables the number of reagents to be set, resulting in getting the assumed nanostructure sizes inside the silica pores.

The initial materials for presented nanocomposites were as follow:

- SBA-15 silica containing propyl copper phosphonate units (hereafter called SBA-POO_2_Cu) for copper pyrophosphate nanocrystals containing nanocomposite and
- SBA-15 silica containing propyl silver carbonate units (abbreviated SBA-COOAg) for the specimen with silver oxide nanocrystals.

The syntheses were carried out in an argon atmosphere. The solvents were dried and distilled immediately prior to their use. Reagents with the highest available purity were used for the reactions. Triblock copolymer Pluronic P123 ($EO-20PO_{70}EO_{20}$, where EO poly-ethylene oxide and PO is poly-propylene oxide), tetraethyl orthosilicate (TEOS), butyronitriletriethoxysilane (BNTES), bromotrimethylsilane (BrTMS), chlorotrimethylsilane (ClTMS) and copper acetylacetonate (Cu(acac)_2_) were purchased from Aldrich and used as supplied. Phosphonate propyl dietyltriethoxysilane, hereafter called PPTES, were purchased from Syntal Chemicals.

The initial materials—functionalized SBA-15 mesoporous silicas were prepared according to the direct-synthesis route [24,25], which is based on the co-condensation of TEOS and PPTES (for SBA-POO_2_Cu) or TEOS and BNTES (for SBA-COOAg) in the presence of P123 as the structuring agent under acidic conditions. We show the synthesis route of the initial materials (stage 1) in Figure 2.

We demonstrated how to tune the internal nanocrystal sizes in silica channels by the modification of the functional unit concentration in initial material. For this reason, we prepared smaller nanocrystals of copper pyrophosphate by the use of initial material that contained 5% (molar) of functional units, and larger crystals of silver oxide using of the initial material containing 10% of functional groups. To correctly define the doping rates, we set the molar ratios between TEOS (precursor of silica units) and PPTES or BNTES (precursors of functional units). To have a doping rate of 5% (for SBA-POO_2_Cu) we took one moll of PPTES for 19 molls of TEOS (the N in Figure 2a is equal to 19), while, for doping rate of 10%, we used nine molls of TEOS for each moll of BNTES (the N in Figure 2b is equal to 9).

In the first step (STEP1), the precursors of silica and functional units were mixed in the aqueous solution of HCl with pH = 1.5 and P123 surfactant (160 mL of the aqueous solution of HCl, 4 g of P123 surfactant and 45 mmols of a total of TEOS and PPTES or TEOS and BNTES). After two hours of stirring, 75 mg of NaF were added to this mixture to induce hydrolysis and polycondensation. Next, the materials were immediately heated to 60 °C in a hot oil bath and then stirred at this temperature for 48 h under reflux. The resulting materials were filtered and washed with ethanol. The surfactant was removed via a hot ethanol extraction in a Soxhlet apparatus for eight hours. The resulting materials contained groups of the ester of phosphonic acid or cyano units (see Figure 2).

In this stage, the silica surface also contains numerous surface hydroxyl units, which can react with phosphonic or carbonic acids during hydrolysis, thereby making them inactive. To prevent this, the sylilation was carried out to eliminate surface hydroxyl units. This was done by treating the mesoporous powder with a solution of 9 mmols of ClTMS for each gram of the silica material in toluene (200 mL) under reflux for 12 h. After washing with toluene and drying, the samples were ready for the next processing.

To convert the groups of the ester of phosphonic acid and cyano units to phosphonic acid and carbonic acid groups, hydrolysis was needed (STEP2). The materials were processed in a different way. For the case of SBA-POO_2_Cu, we converted ester units into phosphonic acid by selective two-step hydrolysis by BrTMS [26]. This aim was achieved by mixing the samples with a solution of bromotrimethylsilane in toluene (two equivalent parts of BrTMS for the molar number of the functional units) under reflux for 24 h. Next, we washed the powder with toluene, dried, and put under the reflux once again, this time with methanol. After 5 h, the resulting powder was filtered out and dried. After this procedure sample contained phosphonic acid groups anchored inside silica pores via propyl chains.

For the case of SBA-COOAg sample, the acidic hydrolysis was applied to convert cyano groups into carbonic acid units. To do this, the silica was mixed with cyano groups with a mixture of concentrated HCl and acetone (120 mL of HCl and 80 mL of acetone) under reflux for 12 h. Acetone is necessary for this process since, after hydrolysis, silica powder is strongly hydrophobic. The addition of acetone facilitates in penetrating the pores’ interior by the solution. After this process, the powder was washed with a mixture of water and acetone (1:1) several times until neutral pH level was reached. The powder was recovered by filtration and dried.

In the STEP3, functionalization was carried out. As with the previous step, the procedure was different for both samples. In order to functionalize the silica containing the phosphonic acid groups using copper ions, we added 0.25 g of Ni(acac)_2_ to each gram of the silica powder (slightly more than necessary 0.75 mmols), and put the mixture under reflux overnight with tetrahydrofuran (about 50 mL for each gram). The resulting solid (SBA-POO_2_Cu) was quantitatively recovered via filtration and washed with THF several times to remove any excess copper salt. After filtration and drying, the initial material—SBA-POO_2_Cu containing 5% of copper phosphonate units (molar)—was ready.

For the case of carbonic acid containing sample, the functionalization was carried out in total darkness to avoid the silver salt degradation. This was done by adding of 0.35 g of AgNO_3_ (slightly more than necessary 16 mmols) to each gram of silica with carbonic acid groups and mixing this in the equimolar composition of deionized water and acetone (water dissolves the silver nitride, while acetone facilitates penetration of the pores interior) under reflux for 12 h. The resulting material was washed with the mixture of water and acetone several times to remove residues of the doping agent. The sample—SBA-COOAg containing 10% of silver carbonate units (molar)—was dried in vacuum for a few hours and stored in total darkness.

In the next stage (stage 2), the initial materials were transformed into nanocomposites containing nanocrystals inside pores. The first sample—SBA-POO_2_Cu—was heated for seven hours at a temperature of 350 °C in the air (heating rate 3 K·min^−1^). After this process, the material (hereafter called SBA-O(POO_2_Cu)_2_ NC) was ready for further investigations. Similarly, SBA-COOAg sample also was heated for seven hours, but, this time, in the temperature of 900 °C in the air (heating rate 3 K·min^−1^). The resulting sample was named SBA-Ag_2_O NC.

Both obtained materials were observed by a transmission electron microscope (FEI Tecnai G2 20 X-TWIN) to confirm the assumed structure. The TEM patterns of the samples before and after thermal treatment, along with electron diffraction patterns, are shown in Figure 3. For fitting the diffraction patterns, we used Eldyf software (property of Institute of Material Science of the University of Silesia).

The TEM observations of the initial materials (SBA-POO_2_Cu—Figure 3a and SBA-COOAg—Figure 3b) confirm that both investigated samples have ordered mesoporous structure, typical for SBA-15 silica. This observation can also suggest that copper-containing functional groups homogeneously distributed over space inside pores (no bulky agglomeration of doping agent are observed). The electron diffraction pattern confirms amorphous structure, according to assumption. In-depth characterization of such kinds of materials can be found in our other works [27,28].

The structure of the target samples—nanocomposites containing nanocrystals inside silica pores—differs significantly depending on the sample. For the case of the copper-containing material, after calcination of the initial material in the air for seven hours at 350 °C, the nanocrystals appear inside silica pores. They are visible under the TEM microscopy after the close inspection, especially in the dark field (SBA-O(POO_2_Cu)_2_ NC—Figure 3a). The nanometric crystals have a spherical form with a diameter of below 3 nm, and they are placed inside silica channels, as it can be clearly seen in the magnified TEM images, presented in Figure 4, left side. The electron diffraction pattern confirms the crystalline structure of the nanoobjects located inside the pores. Moreover, fitting the diffraction rings by theoretical model pointed out the copper pyrophosphate structure (see Figure 3a bottom row at the right side) [29]. We identified the characteristic diffraction rings from the planes of 200, 011, 11-1, 210, 120, 121, 22-1, 400. Additional X-Ray diffraction analysis, confirming the structure can be found in Supplementary Materials (see: Figure S1a).

As far as the silver-containing sample is concerned (SBA-Ag_2_O NC—Figure 3b), similarly, in this case, initial material has a proper structure with no bulky agglomeration, and after calcination the nanocrystals appear inside silica channels. This time also crystalline structure of the internal nanoobjects was confirmed by the electron diffraction pattern. Fitting diffraction rings to the electronogram verified positively the assumed structure of Ag_2_O on the basis of presence of diffractions originating from 110, 200, 310, 422 planes (see also Supplementary Materials for XRD analysis—Figure S1b) [30]. However, in this case, nanocrystals are significantly larger and sometimes have the shape of short rods instead of spheres (see: Figure 4, right side). Moreover, some bigger crystals/crystals agglomerations are also visible (see: Figure 4, right side, green-marked structures). Probably, such structures are placed on the surface of the silica carriers, but there is a chance for the modification of the whole SBA-15 structure. This can be also a result of the light impact during the functionalization procedure (we prevented from this, but we cannot exclude such a case). One must remember, nevertheless, that the silver-containing initial material (SBA-COOAg) possessed two times more functional units than previously described sample: SBA-COOAg had 10% of functional units (molar proportions to silicon atoms), while SBA-POO_2_Cu only 5%. As can be clearly seen, the size of internal nanocrystals depends on the functionalization rate and can be tuned by modification of the number of functional units. After some kind of calibration of the initial materials, we can fabricate the assumed size of nanocrystals for some specific applications. Of course, they are limited to the diameter of silica channels (some structures larger, than the size of pores can be placed on the surface of the silica support). For this sample it is clearly seen that the SBA 15 channels size have a limiting effect on the crystalline grown. Furthermore, looking at the electron diffraction pattern it seems that Ag_2_O crystals are oriented. This effect can takes from two factors: some relatively large nanocrystal can have a dominating signal (since we can find relatively large crystals in the material), or the porous silica matrix involves some orientation of the crystal growth. We must remark that for the case of SBA-Ag_2_O NC the sizes of crystals are not so uniform, as for the previous material. Probably the method is more effective for the copper-containing material.

Both materials presented above were designed for various applications. Numerical simulations suggest that the nanocomposite containing copper pyrophosphate nanocrystals in silica channels (SBA-O(POO_2_Cu)_2_ NC) can be treated as a layout of semiconducting quantum dots (SQD) [31,32]. The silica matrix provides an ambient environment for the spatially confined crystalline object. The size of the separated nanocrystals is below 5 nm (close to 3 nm). Moreover, our DFT-determined density of states (DOS) diagram (QuantumEspresso software) suggests the semiconducting-like properties of copper pyrophosphate material. It is seen in Figure 5 that the electronic states near the top of the valence-band are mainly filled by Cu-d and O-p electrons localized in the energy range from −5.0 eV almost to the Fermi level. Furthermore, the spin-polarized calculation shows the presence of a narrow peak in the spin-up channel and slightly broader one in spin-down channel both concentrated around Fermi energy. Closer inspection of DOS spectra also reveals an additional peak above the Fermi level in the spin-down channel, and this feature is formed by the Cu-d and O-p states.

This output can point out that the copper pyrophosphate nanocrystals inside a porous silica matrix can be treated as SQDs. The material, as such, has a great applicative potential in photonics and quantum computations. The non-linear optical properties of the system presented here, its structural and electronic properties supported by thorough numerical simulations are a subject of separate papers.

The nanocomposite with silver (SBA-Ag_2_O NC), in turn, can find an application in medicine as a disinfection agent. The silica plays here an important role of an interface, allowing for the material use as a filler for plastics and mixing it with, for example, paints. Silver nanocrystals are active biocidal centers with a vast active surface area (which results from their small and relatively uniform sizes). The biological properties of this material are described in our other article (under review).

In summary, we have shown the procedures allowing for fabrication of the nanocomposites composed of nanocrystals placing inside the SBA-15 silica channels. The presented synthesis routes allow for tuning the sizes of internal crystals and setting its chemical composition by treating the silica pores as nanoreactors. The direct-synthesis method facilitates homogenous functionalization of the silica channels interior with assumed elements and assumed doping rate [27,28]. Such an initial functionalized porous material undergoes the thermal decomposition, resulting in the creation of the internal nanocrystals. The synthesized nanostructures have dimensions depending on a doping rate of initial material and the structure being derivative of the functional unit type. The procedure allows for obtaining the compound with the precisely tailored application for some specific application. The functionalization procedures presented here can be easily generalized for the preparation of the materials with other types of internal nanocrystals for the applications different than quoted here.

## Figures and Tables

**Figure 1:**
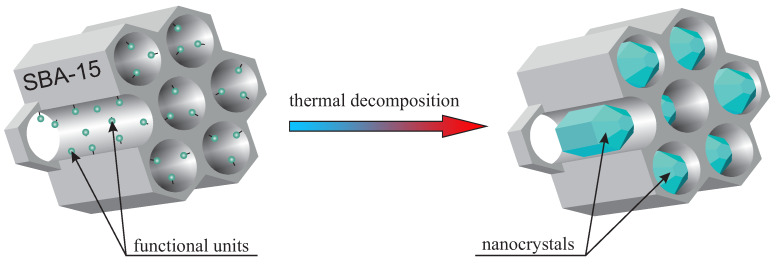
Visualization of the procedure of nanocrystals fabrication in silica nanoreactor.

**Figure 2:**
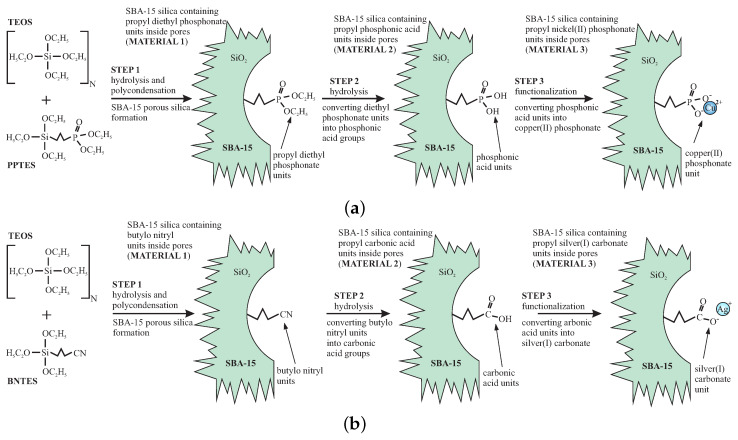
Schematic presentation of the synthesis route of the initial materials: SBA-15 silica containing propyl copper phosphonate units (**a**) and SBA-15 silica containing propyl silver carbonate units (**b**). The number of N defines the doping rates by setting proportions between precursors of silica and functional units.

**Figure 3:**
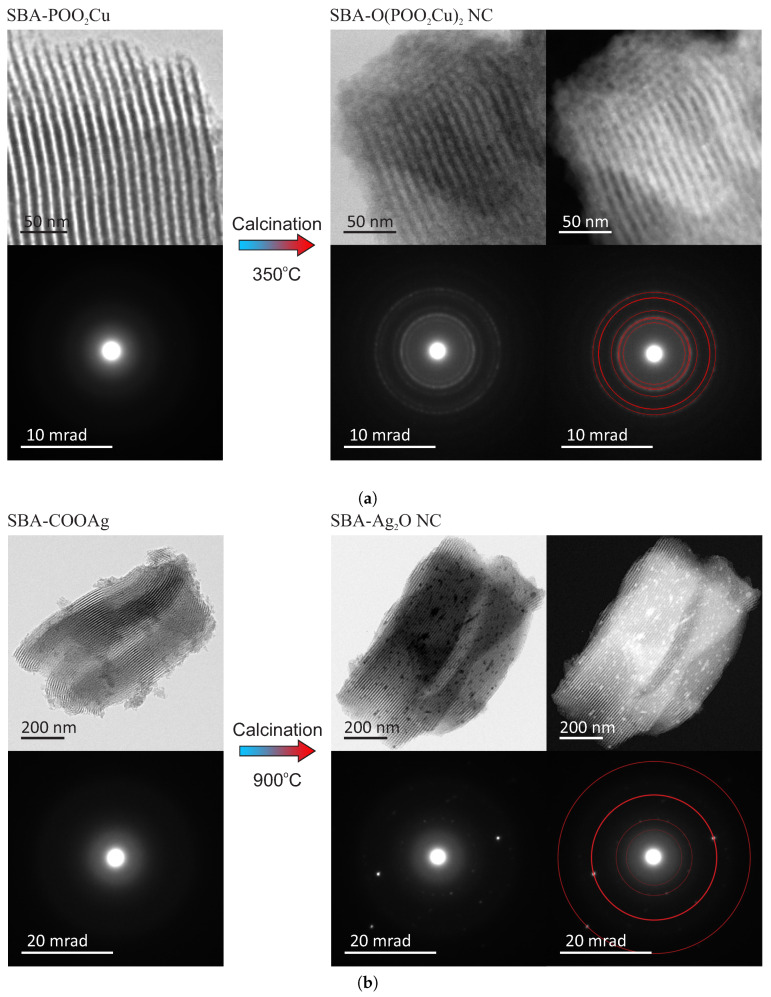
TEM images of the investigated materials (upper rows) along with the electronograms (bottom rows): copper-containing material (**a**) and silver-containing ones (**b**). At the left side we can see the initial material (silica containing anchored metal ions—after stage 1 of the synthesis), while at the right side target material is presented (silica containing nanocrystals inside pores—after stage 2 of the synthesis: calcination at the high temperature). Both target materials were captured at light (left side) and dark field (right side) to make crystals well-visible. The bottom rows show electron diffraction patterns for the samples; the target samples show visible crystalline pattern, thus we present electronograms both native and with fitted theoretical diffraction rings for the copper pyrophosphate structure (**a**) and Ag_2_O crystal (**b**).

**Figure 4:**
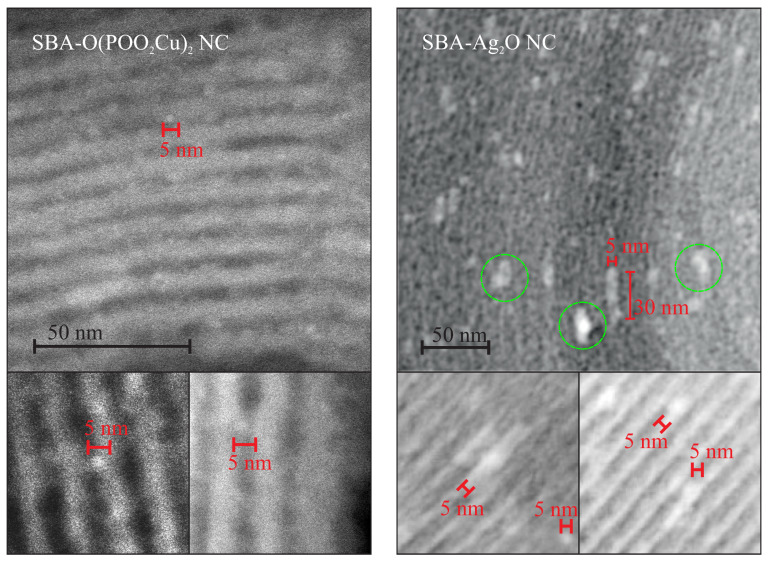
High magnification TEM images of the investigated materials with the markers allowing for estimation of the nanocrystals size. Additional magnified figures with increased contrast and more markers can be seen at the bottom row. The green circles in the TEM image of silver-containing material pointing out the crystal agglomerations, probably on the surface of the silica carrier.

**Figure 5:**
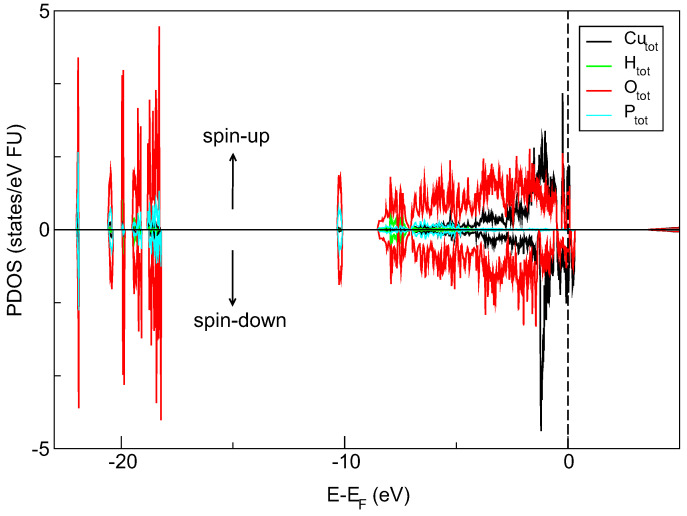
Spin resolved density of states (DOS) with partial atomic contributions in copper pyrophosphate material calculated with the PBE functional. The vertical dashed line shows the position of the Fermi level.

## Supplementary Materials

The following are available online at https://www.mdpi.com/1996-1944/13/9/2009/s1, Figure S1: X-ray diffraction pattern for the investigated samples: silica-based nanocomposites containing copper pyrophosphate—SBA-O(POO2Cu)2 NC (a) and silver oxide—SBA-Ag2O NC (b) nanocrystals inside pores. Under the spectrum, characteristic theoretical reflexes are presented as bars. Indexes that can be assigned to the peaks are presented at the spectrum, while those invisible at the theoretical bars.

## Abbreviations

The following abbreviations are used in this manuscript:

TEOS	tetraethoxy silane
BNTES	butyronitriletriethoxysilane
PPTES	phosphonate propyl dietyltriethoxysilane
BrTMS	bromotrimethylsilane
ClTMS	chlorotrimethylsilane
SQD	semiconducting quantum dots
DOS	density of states
