# Supplementary material for: Synthesis in Silica Nanoreactor: Copper Pyrophosphate Quantum Dots and Silver Oxide Nanocrystallites Inside Silica Mezochannels
Source: Materials (Basel). 2020 Apr 25;13(9):2009. doi: 10.3390/ma13092009 (PMC7254358; doi:10.3390/ma13092009)
Supplement: Supplementary file 1 [file materials-13-02009-s001.pdf]

materials

MDPI

Supplementary Materials

Synthesis in Silica Nanoreactor: Copper Pyrophosphate Quantum Dots and Silver Oxide Nanocrystallites Inside Silica Mezochannels

Additional experiments

X-Ray diffraction

The XRD patterns were measured using Bruker D8 Advance diffractometer (Billerica, Ma, USA) with CuKα radiation (λ = 1.5418 Å) and LynxEye detector, operating at 40 kV and 40 mA. The studies were performed in conventional Bragg-Brentano configuration for the range of 2θ from 10° to 100° with step size of 0.01° and step time of 5 s. For the analysis of the peak, we used Match! Software (V1.11).

The powder XRD spectra of the porous silica-based nanocomposites containing copper pyrophosphate (SBA-$O(POO_2Cu)_2$ NC) and silver oxide (SBA-Ag_2O NC) nanocrystals inside pores can be seen in Figure S1.

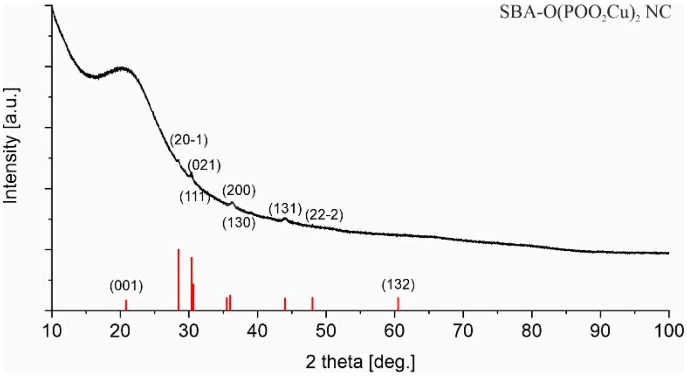

(**a**)

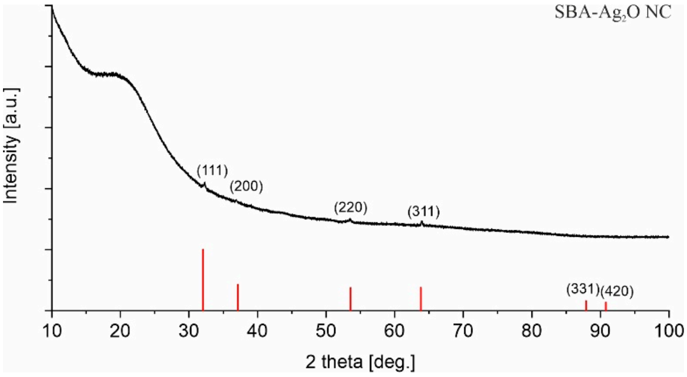

(**b**)

Figure S1. X-ray diffraction pattern for the investigated samples: silica-based nanocomposites containing copper pyrophosphate–SBA-$O(POO_2Cu)_2$ NC (**a**) and silver oxide–SBA-Ag_2O NC (**b**) nanocrystals inside pores. Under the spectrum, characteristic theoretical reflexes are presented as bars. Indexes that can be assigned to the peaks are presented at the spectrum, while those invisible at the theoretical bars.

The samples presented the main features characteristic for amorphous SBA-15 type silica: a broad peak at the 2theta of about 20° [1]. Above it, also Bragg peaks originating from the crystalline structure was visible. The crystalline peaks were very week, however. It is typical for such small

nanocrystals. We identified the types of internal crystals by the comparison of the peaks with crystalline database (Match! Software). Matching the structure confirmed, that we observe copper pyrophosphate nanocrystals inside SBA-$O(POO_2Cu)_2$ NC sample, and silver oxide – Ag2O – inside SBA-Ag_2O NC sample. Almost all most significant Bragg peaks (peaks for the intensity at least 20% of maximum) were visible at the spectra. For the case of SBA-$O(POO_2Cu)_2$ NC material (001) and (132) reflexes were not visible. It was probably caused by their low intensity and the overlapping signal from SB-15 structure ((001) peak for SBA-$O(POO_2Cu)_2$ NC sample).

The Rietveld analysis and estimation of the nanocrystals sizes were not possible. Such a week, crystalline peaks caused a very high calculation error, much higher than the dimensions of the crystals.

© 2020 by the authors. Submitted for possible open access publication under the terms and conditions of the Creative Commons Attribution (CC BY) license (http://creativecommons.org/licenses/by/4.0/).
